# Transfer delays in patients referred for neurosurgical intervention with traumatic brain injury

**DOI:** 10.1186/cc11082

**Published:** 2012-03-20

**Authors:** L Smith, B Jordan, J Paddle

**Affiliations:** 1 Royal Cornwall Hospital NHS Trust, Truro, UK; 2 Derriford Hospital, Plymouth, UK

## Introduction

National guidance for patients presenting to the emergency department (ED) with a traumatic head injury advises that head computed tomography (CT) should be performed and reported within 1 hour [1]. The operative intervention or injury to knife time should be within 4 hours [2]. With more than 50% of patients requiring neurosurgical intervention in the UK taken to hospitals without onsite neurosurgical services [3], secondary transfer is necessary prior to definitive intervention. Are we achieving timely transfers in rural England?

## Methods

The Royal Cornwall Hospital is a district general hospital serving a population of 300,000. The regional neurosurgical unit is 100 km away. All patients undergoing transfer to the neurosurgical unit during 2009 were identified. A notes review was undertaken of all these patients transferred to the care of neurosurgeons. The operative logs were also reviewed. Time lines were created of their care from ambulance call to neurosurgical intervention.

## Results

Ten patients in total were transferred for neurosurgical intervention. Two of these patients required two transfers as they were initially seen in satellite minor injury units. No patient had CT within 1 hour of arriving in the ED. The median time was 2 hours 56 minutes. The CT report was available at a median of 3 hours 17 minutes. None of these patients arrived in the tertiary referral centre within 4 hours of their injury. The fastest time to intervention was 8 hours 29 minutes, median 22 hours 59 minutes after injury.

## Conclusion

We are not meeting targets for CT head acquisition and transfer for neurosurgical intervention. Prompt transfer of a trauma patient from a rural district general hospital in the UK to a tertiary referral centre for neurosurgical intervention is a multifactorial problem. The introduction of trauma centres and of protocols for direct admission to tertiary centres by paramedics may reduce the delays that our audit has highlighted.
